# Application of a Combined Homogenate and Ultrasonic Cavitation System for the Efficient Extraction of Flavonoids from *Cinnamomum camphora* Leaves and Evaluation of Their Antioxidant Activity *In Vitro*

**DOI:** 10.1155/2019/4892635

**Published:** 2019-02-07

**Authors:** Zaizhi Liu, Lingtao Kong, Shunbao Lu, Zhengrong Zou

**Affiliations:** College of Life Sciences, Jiangxi Normal University, Nanchang 330022, China

## Abstract

A free-of-dust pollution extraction method combined-homogenate and ultrasonic cavitation system, namely, homogenate-combined ultrasonic cavitation synergistic extraction (HUCSE), was proposed for the efficient extraction of flavonoids from *Cinnamomum camphora* leaves. Response surface methodology of Box–Behnken design was employed to optimize the HUCSE process, and the optimum operation conditions attained with an extraction yield of 7.95 ± 0.27 mg/g were ethanol concentration 76%, homogenate/ultrasonic time 25 min, solvent-to-solid ratio 22 mL/g, and ultrasonic power 240 W. A second-order kinetic mathematical methodology was performed to depict the behaviors of HUCSE and heat reflux extraction method. The results suggested that the developed HUCSE is an efficient and green method for the extraction of *C. camphora* flavonoids or other plant natural products, where the obvious higher parameters of extraction capacity at saturation, second-order extraction rate constant, and original extraction rate were obtained when compared to the heat reflux method. The antioxidant activity assays *in vitro* showed that the *C. camphora* flavonoids possessed strong antioxidant activity and are promising to be applied as a natural alternative antioxidant.

## 1. Introduction

Natural products have gained great interest as natural alternatives to substitute the traditional synthetic compounds for health purposes as their remarkable health-promoting activity and consumer preference for the cleaning labels [1, 2]. Specially, natural products even have been considered as an abundant source for drugs production, thanks to their chemical structures diversity that synthetic compounds cannot compare [3]. As an important member of plant natural products, flavonoids are commonly occurring in nature with variant phenolic structures [4]. Flavonoids are well known for their versatile health benefits that mainly ascribe to their antioxidant activity [5], anti-inflammatory activity [6, 7], and antitumor activity [8–10].

*Cinnamomum camphora* is an evergreen tree, which is widely distributed in Southern China as a Chinese folk medicine for the cure of many diseases [11]. *C. camphora* is even extensively cultivated in Jiangxi province as a landscape tree species and an economic crop for the production of essential oil and camphor [12]. Many recent studies which highlighted the utilization of *C. camphora* are mainly on essential oils used for their antifungal and insecticidal activities [13–15], and the other bioactive compounds are rarely involved. Notably, previous studies demonstrated that flavonoids are indwelling in *C. camphora* leaves with remarkable antioxidant activity and antibacterial activity [16–18]. Moreover, a previous study suggested that scientific harvesting of *C. camphora* leaves at regular intervals facilitates *C. camphora* to grow vigorously [19], so it is an ideal source for the separation of flavonoids.

Generally, plant materials are ground to powders using mechanical disintegrators prior to the subsequent extraction process for sufficiently obtaining the target compounds. Among the physical smashing ways, homogenate is an effective technique as it possesses many inherent merits (e.g., high-speed mechanical shearing effect, agitating, pulverization, and no powder pollution), which is conducive to the dissolution of plant bioactive compounds into extraction solvent [20, 21]. Extraction of flavonoids is traditionally attained by techniques involving heating, boiling, or refluxing. However, these techniques have been pointed out to be subjected to the shortcomings of low extraction efficiency on account of hydrolyzing, ionizing, and oxidation reaction during the natural bioactive compounds extraction processes as well as large volume of solvent consumption and long extraction time consumption [21–24]. In this respect, an environmentally friendly process associated with the maximization of the target compounds yields with minimum degradation, resulting in the most effective constituent at a low cost would be ideal [25]. Innovative techniques on the basis of ultrasonic-assisted extraction have been employed to separate plant natural products and have showed great potential on obtaining high valuable target constituents [26–29]. The proposed principle of the ultrasonic-assisted extraction method is sonochemistry and mechanical effects induced by ultrasonic cavitation. For one thing, high strength ultrasonic waves can cause pressure fluctuations when they propagate through liquid medium which can instantaneously give rise to plenty of vacuum-filled cavitation bubbles [30]. These bubbles are highly fragile and unstable and can implode abruptly within a few milliseconds. Accompanying with the collapse of cavitation bubbles, local temperature and pressure will increase, which favors for solvent circulation and penetration within the cellular plant materials as well as improving mass diffusion rate, thus enhancing the extraction performance [31]. In addition to the cavitation phenomena of sonochemistry, the mechanical effect generated by microstreaming and microturbulence facilitates cell walls breaking mechanically and hence promoting the release of target compounds from the plant materials into the extraction medium [32]. Apart from the merits in decreasing extraction time, saving energy, and improving the extraction yields, easy operation, and low cost, the remarkable benefit of ultrasonic-assisted extraction is that it is beneficial to thermolabile target compounds extraction with almost no degradation [33]. Thus, developing a reliable, highly efficient, and green extraction method-combined homogenate and ultrasonic cavitation for obtaining *C. camphora* flavonoids is much necessary.

The response surface method (RSM) is an efficient statistical optimization methodology which just needs a few numbers of experimental trials to investigate multiple variables and their interactive effects [34]. RSM is useful for optimizing complex procedures in which response values of interest are affected by various parameters, and the objective is to obtain the optimal conditions and the desired yield; thus, it has gained great interest to be widely employed in optimizing plant natural products extraction processes [35–38]. In this study, a simple, effective, and no-dust pollution homogenate-combined ultrasonic cavitation synergistic extraction (HUCSE) method was proposed for the extraction of flavonoids from *C. camphora* leaves. An RSM-based Box–Behnken design (BBD) was applied to offer the optimal combination of ethanol concentration, homogenate/ultrasonic time, solvent-to-solid ratio, and ultrasonic power with which a maximum yield of *C. camphora* flavonoids can be attained by HUCSE and their antioxidant capability can be evaluated *in vitro*.

## 2. Materials and Experimental

### 2.1. Reagents and Materials

Chromatographically pure rutin, 2,2-diphenyl-1-picrylhydrazyl (DPPH), vitamin C (V_C_), butylated hydroxyanisole (BHA), butylated hydroxytoluene (BHT), and 2,4,6-*tripyridyl*-s-triazine (TPTZ) were obtained from Aladdin Reagent Co. (Shanghai, China). Other analytically pure reagents were obtained from Beijing Chemical Reagents Co. (Beijing, China). Deionized water used in the experiments was purified by a Milli-Q water system (Millipore, Waltham, MA, USA). *C. camphor* leaves were collected in November 2017 from Jiangxi Normal University campus (Jiangxi, China) and authenticated by Prof. Ronggen Deng (Jiangxi Normal University, China). Fresh leaves were dried at room temperature (20 ± 0.8°C) for 7 days, stored in a nylon bag, and then placed in a dry and ventilated place before the subsequent experiments.

### 2.2. Apparatuses

The HUCSE apparatus was employed for *C. camphora* flavonoids extraction as described in our previous study [25], which is a self-assembling equipment comprising a Philips disintegrator (Guangdong, China) and a Biosafer250up handheld Sonifier cell disrupter (SaferCo. Ltd, China). The highest shearing speed and volume of the disintegrator are 10,000 r/min and 250 mL, respectively. The Sonifier cell disrupter power can be regulated freely with a range of 0–250 W.

### 2.3. Quantification of Flavonoids by UV-Vis Spectrophotometry

The quantification of *C. camphora* flavonoids was conducted using the aluminum chloride colorimetric determination method according to Wang et al. [39] with slight modifications. 1 mL diluted rutin standard solution (prepared using 60% ethanol aqueous solution) or sample solutions were transferred into 10 mL volumetric flasks and 1 mL of AlCl_3_ solution (5%) and 2 mL of CH3COONa solution (1 mol/L) were added, followed by adjustment to the scale with 60% ethanol aqueous solution. The mixtures were placed for 20 min in atmospheric temperature and then detected at 400 nm against the mixture without coloration as a reference by a UV-Vis spectrophotometer (751-GW, Shanghai, China). The yields of the flavonoids were conveyed as milligram of rutin equivalents per gram of dry weight of *C. camphora* leaves based on the standard calibration curve. The calibration curve (*y* = 2.40*x* − 0.0645, where *y* is absorbance at 400 nm, *x* is the concentration value) ranged from 0 to 0.05 mg/ml (*R* ^2^ = 0.9963). All experiments were conducted three times, and the results were conveyed as mean ± SD.

### 2.4. HUCSE Process

Dried *C. camphora* leaves were weighed and transferred into the HUCSE system accompanied with the addition of a definite volume of ethanol solution followed by treatment under the presetting conditions for flavonoids extraction. After each treatment, the suspension was centrifuged at 8,000 × g for 20 min to collect the crude extract solutions and then stored in a refrigerator prior to the analysis of flavonoids content by UV-Vis spectrophotometry. Each experiment trial was performed in triplicate for accuracy.

### 2.5. Experimental Design

BBD, a member of RSM, is characterized with a spherical and revolving design. It comprises a central point, and the intermediate points circumscribed on the sphere at the cube edges [40]. Herein, a three levels of four parameters of BBD with a total of 29 experiment trails (24 factorial points and 5 replicates of the central points) was employed to investigate the independent and interactive influences of the four factors on the extraction yield of flavonoids, and the factors are ethanol concentration (*A*, %), homogenate/ultrasonic time (*B*, min), solvent-to-solid ratio (*C*, mL/g), and ultrasonic power (*D*, W). The scope and level of each factor as shown in Table 1 are gained based on the single factor experiments. All the experiment trials generated from Design-Expert software were performed in triplicate in randomized order, and the average value from three replicates of each experiment trial was recorded as the response value.

The Design-Expert (Version 8.0, Minneapolis, USA) software was employed for performing the model establishment, experimental design, analysis of data, and graph plotting. A quadratic equation was fitted to investigate the correlation between the determined values and the four independent variables as follows:

(1) $$\begin{array}{c} Y=\beta_{0}+\overset{4}{\underset{i=1}{\sum }}\beta_{i}X_{i}+\overset{4}{\underset{i=1}{\sum }}\beta_{ii}X_{i}^{2}+\overset{3}{\underset{i=1}{\sum }}\overset{4}{\underset{j=i+1}{\sum }}\beta_{ij}X_{i}X_{j}, \end{array}$$

where *β*_0_ is presented as the constant; *β*_i_, *β*_ii_, and *β*_ij_ are defined as the coefficients of linear, quadratic, and cross-product, respectively. *X*_i_ and *X*_j_ are given as the independent factors at different levels.

### 2.6. Conventional Extraction Method

The conventional heat reflux method (HRE) was performed as the reference method to make a comparison with the proposed HUCSE [41]. HRE was carried out three times under a fixed operation power of 1 kW for 4 hours using an electric jacket. 10 g of dried *C. camphora* leaves powders (60 meshes of particle size) was weighted and placed into a glass flask, and then a definite volume of ethanol aqueous solution was added before subjecting to extraction by HRE. The other extraction conditions applied in HRE were the optimal conditions based on the results of the optimizing process for HUCSE. After the extraction, the mixture was transferred to certification and then analyzed by UV–Vis spectrophotometry as mentioned above.

### 2.7. Kinetic Model

The second-order kinetic model offers an adequate 1 acceptable illustration with respect to the solid-liquid extraction procedures[42] and has been used prominently in modeling extraction [43, 44]. It generally divides the whole extraction process into two coinstantaneous stages: (1) at the beginning the extraction yield rises rapidly over time, and (2) the extraction yield decreases slowly with the increase in time till to the end of extraction [45]. The extraction kinetics for flavonoids from *C. camphora* leaves by HUCSE and HRE were investigated on the basis of the second-order kinetic model. The model parameters *h*, *k*, *Y*_*s*_, and *Y*_*t*_ are defined as the original extraction rate (mg/g·min), second-order extraction rate constant (mg/g·mim), extraction capacity (mg/g) at saturation, and the extract yield (mg/g) at any moment *t* (min), respectively. The dissolution rate of flavonoids contained in the solid transferring to solvent can be represented as

(2) $$\begin{array}{c} \frac{dY_{t}}{d_{t}}=k{\left(Y_{s}-Y_{t}\right)}^{2}. \end{array}$$

Considering the boundary conditions from *t* = 0 to *t* and the corresponding *Y*_*t*_ = 0 to *Y*_*t*_, the second-order model equation (1) can be integrated and rearranged into equation (2) as follows:

(3) $$\begin{array}{c} Y_{t}=\frac{Y_{s}^{2}kt}{1+Y_{s}kt}. \end{array}$$

When equation (3) is linearized, it can be expressed as

(4) $$\begin{array}{c} \frac{t}{Y_{t}}=\frac{1}{kY_{s}^{2}}+\frac{t}{Y_{s}}. \end{array}$$

On arranging equation (3), the extraction rate can be calculated using equation (4):

(5) $$\begin{array}{c} \frac{Y_{t}}{t}=\frac{1}{\left(1/kY_{s}^{2}\right)+\left(t/Y_{s}\right)}. \end{array}$$

The initial extraction rate (*h*) can be denoted by equation (6) when *Y_t_* = *t* at a time of *t* approach O:

(6) $$\begin{array}{c} h=kY_{s}^{2}. \end{array}$$

On arranging equation (4), we get the following equation:

(7) $$\begin{array}{c} \frac{t}{Y_{t}}=\frac{t}{Y_{s}}+\frac{1}{h}. \end{array}$$

The three second-order kinetic model parameters (*h*, *k*, and *Y*_*s*_) can be calculated experimentally by the intercept and slope of the *t*/*Y*_*t*_ versus *t* plot.

### 2.8. Antioxidant Activity Assays

#### 2.8.1. DPPH Radical-Scavenging Assay

DPPH-scavenging test was performed to investigate the free radical-scavenging ability of *C. camphora* flavonoids based on Yang et al. [46]. A series of diluted sample solutions were blended with 3.9 mL 25 mg/mL of DPPH ethanol solution and then placed in dark for half an hour at ambient temperature. The mixed solution absorbance was detected at 517 nm. V_C_, BHT, and BHA were employed to replace the sample solution as positive control groups to make a comparison of *C. camphora* flavonoids. DPPH-scavenging capacity can be determined with the following equation:

(8) $$\begin{array}{c} SC\%=\frac{A_{0}-A_{1}}{A_{0}}\times 100\%, \end{array}$$

where *A*_0_ and *A*_1_ are denoted as the absorbance of the negative control group without sample solution and the tested groups, respectively.

#### 2.8.2. Ferric-Reducing Antioxidant Power (FRAP)

According to the study of Benzie and Strain [47], the antioxidants in attendance can result in reducing of Fe^3+^-TPTZ into Fe^2+^-TPTZ. FRAP solution was comprised of 25 mL 300 mM of acetate buffer solution, 2.5 mL 10 mM of TPTZ, and 2.5 mL 20 mM of FeCl_3_ solution and then subjected to incubation for 30 min around 37°C. The reaction was initiated through mixing 2.85 mL of FRAP solution with 0.15 mL of diluted sample solution and then placed the mixture in darkness for 30 min. After reaction, the absorbance of the mixture was detected at 593 nm using a spectrophotometer. Positive control groups were carried out as above steps by replacing sample solution by V_C_, BHT, and BHA. The ferric-reducing power of flavonoids was conveyed as *μ*M trolox equivalents (TE)/g extracts.

#### 2.8.3. Determination of Reducing Power

The assay for the evaluation of reducing power was performed based on Oyaizu's study [48]. 1 mL of sample liquid, 2.5 mL 0.2 M of phosphate buffer (pH 6.6), and 2.5 mL 1% of K_3_Fe(CN)_6_ solution were mixed and incubated at 50°C for 20 min and then 2.5 mL 10% of trichloroacetic acid was added, followed by centrifugation at 10,000 × g for 10 min. Aliquots of 2.5 mL of the upper layer were collected and then blended with 2.5 mL of deionized water and 0.5 mL 0.1% of FeCl_3_ and then were detected at 707 nm. Positive control groups were carried out as mentioned above by replacing sample solution by V_C_, BHT, and BHA.

### 2.9. Statistical Analysis

ANOVA was used to analyze the statistical significance of all data. BBD was performed by Design-Expert 8.0 software (Stat Ease Inc., Minneapolis, USA), and the actual responses were the average values of each experiment trial in triplicate. The other experiments were conducted in three replicates, and the results are presented as the mean ± SD. OriginPro 2017 software was employed to fit the extraction kinetic curves for HUCSE and HRE according to the second-order kinetic model.

## 3. Results and Discussion

### 3.1. Single Factor Experiments

#### 3.1.1. Influence of Ethanol Concentrations

**Figure 1:**
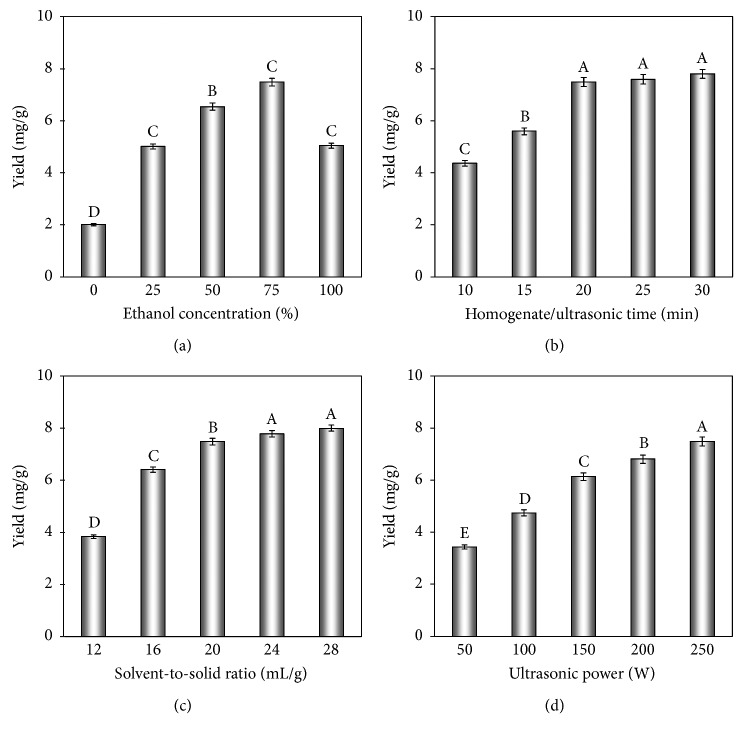
Effect of ethanol concentration (a), homogenate/ultrasonic time (b), solvent-to-solid ratio (c), and ultrasonic time on the flavonoids yield from *C. camphora* leaves (d).

Ethanol is a generally used extractant owing to its remarkable penetration capacity into the plant materials, relatively low boiling temperature (easily recycled) and cost, safety, and nontoxicity [49]. The proportion of ethanol is an important parameter as the extraction yields are largely dependent on the solubility of flavonoids in ethanol solution. Higher proportion of ethanol could result in the dissolution of some liposoluble constituents, but lower proportion of ethanol may bring in incomplete extraction. *C. camphora* leaves (1 g dry weight) were extracted by HUCSE at an ultrasonic power of 250 W for 20 min with various proportions of ethanol (0–100%), and the solvent-to-solid ratio was 20 mL/g. As shown in Figure 1(a), in the rise of ethanol concentration (from 0 to 75%), the extraction yield of *C. camphora* flavonoids showed an increasing tendency. However, an upward trend was observed in the extraction yield when the ethanol concentration was over 75%. So, 75–100% of ethanol concentration was applied for the following experiments as an apparent change of extraction yield was observed in this range.

#### 3.1.2. Influence of Homogenate/Ultrasonic Time

To obtain the appropriate homogenate/ultrasonic time, a series of experiments were conducted at different HUCSE times, and the results are presented in Figure 1(b). The extraction yield was very low at the first 10 min of HUCSE process, which indicated that the synergistic effect of homogenate and ultrasonic cavitation energy needs time to break down the structures of plant cell walls and thus promoting the target analytes released from plant materials into the solvent. It indicated the extraction yield of flavonoids enhanced dramatically with homogenate/ultrasonic time increasing from 10 to 20 min, while long homogenate/ultrasonic time did not result in apparent improvement, and thus 20 min was selected as the middle level. Therefore, a range of 15–25 min homogenate/ultrasonic time was determined for the latter experiments.

#### 3.1.3. Influence of Solvent-to-Solid Ratio

The solvent-to-solid ratio is a crucial factor in the extraction procedure, excessive volumes of the solvent could give rise to the tedious extraction process and needless waste, and minute volumes of solvent may result in the incomplete separation. Hence, different solvent-to-solid ratios from 12 to 28 mL/g with 4 mL/g intervals were studied to analyze their effects. As presented in Figure 1(c), the extraction yield of flavonoids enhanced apparently in the rise of solvent-to-solid ratio from 12 to 20 mL/g, and this is due to the higher solvent-to-solid ratio; the sample could contact solvent more sufficiently, and hence, the extraction yield were improved. With solvent to solids in the excess of 20 mL/g, high volumes of solvent did not trigger in the obvious improvement of the extraction yields. Hence, 20 mL/g was selected as the middle level, and 16–24 mL/g was selected as the suitable range of solvent-to-solid ratio for the further experiments.

#### 3.1.4. Influence of Ultrasonic Power

To analyze the effect of ultrasonic power on the extraction yield of flavonoids, tests were conducted at different ultrasonic powers (50, 100, 150, 200, and 250 W) with 75% of ethanol concentration, 20 min of homogenate/ultrasonic time, and 20 mL/g of solvent-to-solid ratio, respectively. As shown in Figure 1(d), when ultrasonic irradiation power raised from 50 to 150 W, the extraction yield enhanced dramatically; this phenomenon was perhaps because the synergistic influence of the homogenate and ultrasonic cavitation triggered the processes of solvent permeating into the interior plant materials and the target compounds diffusing into solvent occurring more rapidly. Further increase of ultrasonic power just brought a slow improvement in extraction yields; an ultrasonic power around 200 W was sufficient for flavonoids extraction and therefore was chosen as the middle level. Hence, 150–250 W was identified as the suitable range of ultrasonic power.

### 3.2. Experimental Design and Analysis

BBD was used to build the combined influences of four factors on the HUCSE process for *C. camphora* flavonoids extraction and to determine optimal extraction conditions. The extraction yield was defined as the response. The design matrix, numbered, and actual forms at three levels (denoted by +1, 0, and –l) of four independent factors; the determined extraction yields by HUCSE process; and the predicted values generated from BBD are given in Table 1. Ethanol concentration (*A*), homogenate/ultrasonic time (*B*), solvent-to-solid ratio (*C*), and ultrasonic power (*D*) were studied as shown in Table 1. The quality of the developed model was further investigated using ANOVA analysis (Table 2).

#### 3.2.1. RSM Model

The model suitability was verified by the coefficient of determination (*R* ^2^ = 0.9903), the adjusted coefficient of determination (adjusted *R* ^2^ = 0.9754), and the coefficient of variation (C.V = 1.26%), which revealed that more than 99% of the obtained actual values can be interpreted by the selected model, and the model accuracy and generic availability are capable. The predicted *R* ^2^ of 0.9516 was corresponded well with the adjusted *R* ^2^. The adequacy precision estimated the signal-to-noise ratio, and a ratio higher than 4 is generally desirable. The adequacy precision of 38.78 revealed that the developed model could be employed to handle the design space.

The significance of all the coefficients was examined by *F*-test coupled with the *P*-value (Table 2). The developed regression model for the optimization of HUCSE was extremely significant as evidenced by the *F*-value (101.79) and a very low *P*-value (<0.0001) as shown in Table 2. The value of “lack of fit” (0.3111) was insignificant for the developed model, which further affirmed its correctness.

#### 3.2.2. Effect of Process Variables on Extraction Yield

Statistical analysis regarding the regression model for the extraction of *C. camphora* flavonoids suggested that the interactive influences of HUCSE process was extremely significant affected by the ethanol concentration, homogenate/ultrasonic time, and solvent-to-solid ratio and ultrasonic power, in both linear and quadratic manners. The interaction of solvent-to-solid ratio versus ultrasonic power, homogenate/ultrasonic time versus ultrasonic power, and ethanol concentration versus solvent-to-solid ratio or ultrasonic power showed extremely significant, highly significant, and significant influences on the extraction process, respectively. Nevertheless, the interaction effects between homogenate/ultrasonic time to ethanol concentration and solvent-to-solid ratio were statistically insignificant. By using multiple regression analysis on the determined values, the empirical relationship between the extraction yield and four factors in natural values was represented using a polynomial equation:

(9) $$\begin{array}{c} Y=-7.50+0.20\times A+0.15\times B+0.30\times C+0.02\times D+1.03\times 10^{-3}\times AC+1.03\times 10^{-4}\times AD+7.06\times 10^{-4}\times BD+9.70\times 10^{-4}\times CD-1.63\times 10^{-3}\times A^{2}-5.86\times 10^{-3}\times B^{2}-0.01\times C^{2}-1.20\times 10^{-4}\;\times D^{2}. \end{array}$$

#### 3.2.3. Optimization of the HUCSE Procedure

**Figure 2:**
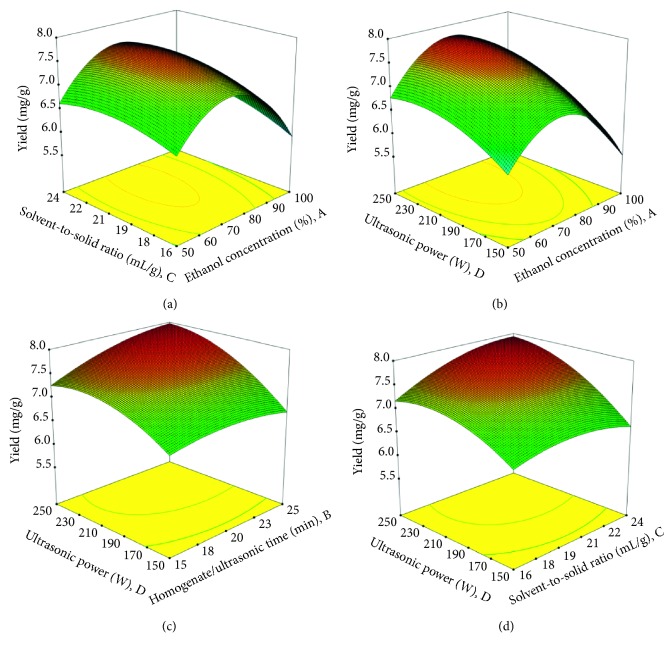
Response surface and contour plots. (a) The reciprocal interaction of ethanol concentration and solvent-to-solid ratio on extraction yield. (b) The reciprocal interaction of ethanol concentration and solvent-to-solid ratio on the extraction yield. (c) The reciprocal interaction of homogenate/ultrasonic time and ultrasonic power on HUCSE process. (d) The interactive effect of solvent-to-solid ratio and ultrasonic power on the extraction yield.

The regression polynomial equation can be delineated by both the three-dimensional response surface and the two-dimensional contour graphs generated from BBD. These graphs offered a way to visualize the connection between response values and detailed experimental levels of four factors and the interactive effects between two test factors on the extraction yield in HUCSE process. The contour plot shapes can reflect the statistical significance of mutual interactions between the factors, with which a circular contour graph indicates that the interactive effect between the factors are insignificant, while an elliptical contour graph suggests that the interactive effect between the factors are significant [50]. The connection between independent and dependent factors was depicted in three-dimensional response surface and two-dimensional contour graphs provided by the developed model for extraction yield; two independent factors were plotted in a three-dimensional surface graph, while the other two factors were maintained at level zero. The three-dimensional response surface and contour graphs illustrating a significant interactive effect of the two factors are presented in Figure 2.

Figure 2(a) shows the reciprocal interaction of ethanol concentration and solvent-to-solid ratio on the yield of flavonoids when homogenate/ultrasonic time and ultrasonic power were fixed at 20 min and 200 W, respectively. It was observed that ethanol concentration demonstrated a quadratic influence, while the solvent-to-solid ratio showed a linear influence on the extraction yield. The extraction yield originally improved with an increase in these two factors and then declined with an increase in ethanol concentration.  Figure 2(b) shows that the reciprocal interaction of ethanol concentration and solvent-to-solid ratio on HUCSE process. Ethanol concentration revealed a quadratic effect, while the solvent-to-solid ratio showed a linear influence on extraction yield.  Figure 2(c) shows the reciprocal interaction of homogenate/ultrasonic time and ultrasonic power on HUCSE process when ethanol concentration and solvent-to-solid ratio were fixed at 75% and 20 mL/g, respectively. The variations of extraction yield were highly significant with an increase in both homogenate/ultrasonic time and ultrasonic power. Likewise, Figure 2(d) shows the interactive effect of solvent-to-solid ratio and ultrasonic power on the extraction yield when ethanol concentration and homogenate/ultrasonic time were fixed at 75% and 20 min, respectively. The reciprocal interaction between solvent-to-solid ratio and ultrasonic power were extremely significant and showed a linear effect on the extraction yield.

#### 3.2.4. Optimization of HUCSE and Verification

As for the BBD optimization process, which needs less experiment trials to be performed for a three-level factorial than other experiment designs to illustrate the significant factors, the possible interactive effects between the variables studied on the extraction yield of target compounds to attain the optimal operation conditions and the satisfied response [51]. By superimposing or overlaying critical response contours on a contour plot, the best compromise can be visually searched. Meanwhile, the function of point prediction allows entering levels for each factor or component into the current model. The software calculates the expected responses and associated interval estimates based on the prediction equation that is shown in the ANOVA output. The optimum HUCSE conditions with a theoretical extraction yield of 8.09 mg/g (ethanol concentration 76%, homogenate/ultrasonic time 25 min, solvent-to-solid ratio 22 mL/g, and ultrasonic power 243 W) for the flavonoids extraction were predicted by BBD of the RSM optimization approach. The abovementioned conditions with slight modifications (ultrasonic power 240 W) were applied to verify experimentally and attain the real extraction yield of flavonoids by HUCSE process. The average extraction yield of flavonoids was 7.95 ± 0.27 mg/g (*n*=3), which was in good accordance with the predicted response by the model equation and further confirmed that the developed response model was capable for the HUCSE process optimization.

### 3.3. Comparison of Extraction Methods and Kinetic Study

**Figure 3:**
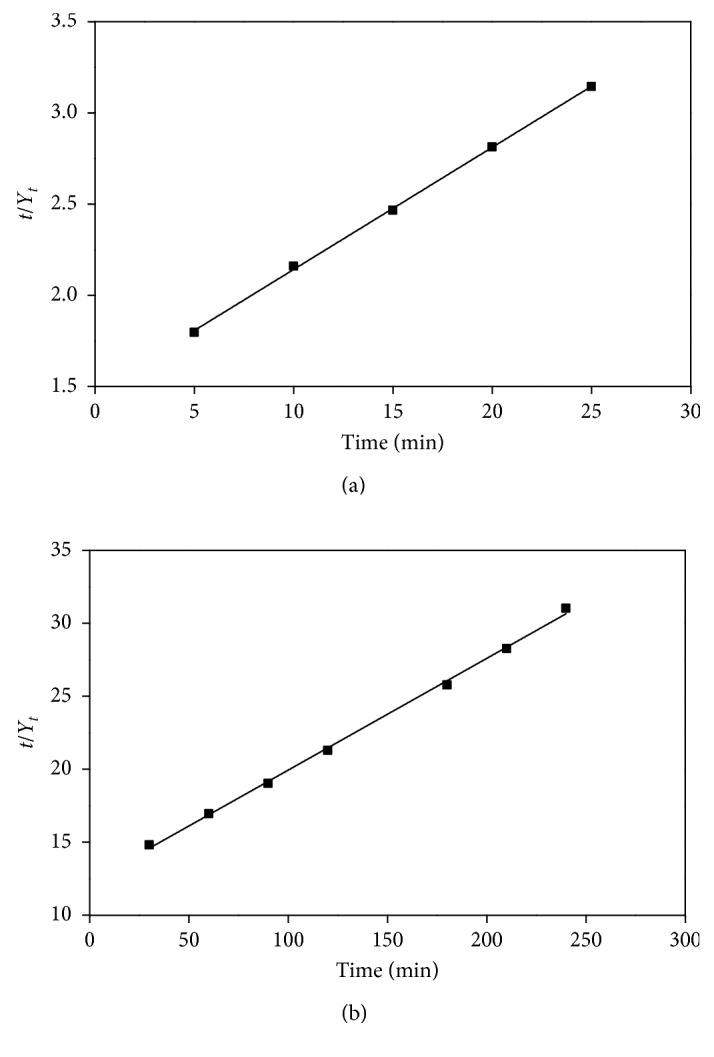
Second-order kinetics curves of HUCSE (a) and HRE (b).

Extraction kinetic modeling was employed to make a comparison of the inherent behaviors of concern on heating and mass transfer during HUCSE and HRE procedures for *C. camphora* flavonoids extraction. The variations of flavonoids extraction yield with changing the treatment time in the procedures of HUCSE and HRE were depicted as presented in Figure 3. HUCSE process obviously saves the extraction time to reach the extraction equilibrium which just spends around one-tenth of the time of HRE. A desirable extraction yield of 7.95 ± 0.14 mg/g was acquired in 25 min by HUCSE, while 240 min of extraction time was consumed by HRE with a maximum extraction yield of 7.72 ± 0.22 mg/g.

The parameters of *Y*_*s*_ (extraction capacity at saturation) and *k* (second-order extraction rate constant) (Table 3) were obtained by fitting the second-order kinetic model and plotting graphs (Figure 3) of *t*/*Y*_*t*_ versus *t* to the actual experimental results. As shown in Table 3, particularly high *R* ^2^ (0.9995 for HUCSE and 0.9984 for HRE) denoted that the second-order kinetic mathematical model was adequate for fitting the HUCSE processes of flavonoids. The parameter *Y*_*s*_ was applied to determine the extraction efficiency of HUCSE compared to that of HRE; it was observed that HUCSE (*Y*_*s*_ = 14.95 mg/g) was more efficient for the extraction of flavonoids than traditional HRE (*Y*_*s*_ = 13.05 mg/g). Meanwhile, the proposed HUCSE possessed apparently higher original extraction rate (*h*) and the second-order extraction rate constant (*k*) for flavonoids separation in comparison to HRE. These results all declared that HUCSE is an efficient technique for the extraction of *C. camphor* flavonoids. It can be concluded that the synergistic effects of the homogenate and ultrasonic cavitation are the main reason that the proposed HUCSE method can dramatically shorten the extraction time and improve the extraction efficiency. A similar result was also observed in our previous study [25].

### 3.4. Antioxidant Activity

**Figure 4:**
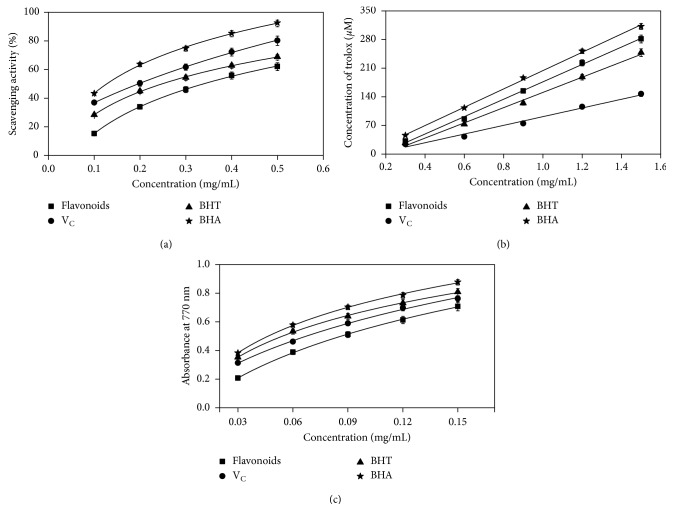
Antioxidant activity of flavonoids from *C. camphora* leaves. (a) DPPH radical-scavenging activity; (b) ferric-scavenging activity; (c) reducing power.

Scavenging activity on DPPH-free radicals is an important indicator on the investigation of antioxidant activity of bioactive compounds [52].  Figure 4(a) reveals the DPPH-scavenging activity of extractant and the control sets with varying concentrations. It was observed a dose dependence manner of *C. camphor* flavonoids on scavenging DPPH radicals. Compared to the positive control sets of V_C_, BHT, and BHA, *C. camphor* flavonoids revealed relatively low DPPH radical-savaging activity at low concentrations, notably with a further increase of concentration, flavonoids showed approximately similar DPPH radical-savaging activity as Vc. FRAP test was performed in the present study to evaluate the antioxidant ability of *C. camphor* flavonoids. As presented in Figure 4(b), linear correlations between the concentrations of extracts or the commercial antioxidants and trolox were observed. Compared to BHA, flavonoids showed a slightly lower reducing ability, while it is adverse to these of VC and BHT and displayed much higher reducing ability at the same concentrations. As shown in Figure 4(c), reducing power assay showed that all four tested compounds exhibited a concentration-dependent tendency on the reducing power. Even if flavonoids enjoyed a little bit lower reducing power than that of VC, BHT, and BHA, the reducing power improved dramatically with the increase of concentration and was very close to that of the three commercial antioxidants. This tendency corresponded well with the results of DPPH radical-scavenging activity. In summary, it can be concluded that *C. camphor* flavonoids showed great potential as a natural antioxidant to substitute the traditional synthetic antioxidants based on the above results.

## 4. Conclusion

In this study, HUCSE technique was developed with the merit of no-dust pollution for the efficient extraction of *C. camphora* flavonoids. BBD was employed to optimize the HUCSE process; the optimal operation conditions were attained and validated, and the results showed that the improved HUCSE method is adequate for the extraction of flavonoids. The second-order kinetic model was performed to investigate the extraction kinetics of HUCSE and HRE of flavonoids, and the kinetic parameters were obtained and analyzed. HUCSE, compared with traditional HRE, enjoyed the higher efficiency for flavonoids extraction and could be extended to isolate other bioactive compounds from plant materials. Additionally, *C. camphora* flavonoids had remarkable antioxidant ability compared to the commercial antioxidants.

## Figures and Tables

**Table 1:** Box–Behnken design matrix and the actual and predicted values for the yields of *C. camphora* flavonoids.

Run	*A*	*B*	*C*	*D*	Yield (mg/g)	
					Actual	Predicted
1	100 (+1)	20 (0)	20 (0)	250 (+1)	6.65	6.70
2	75 (0)	25 (+1)	24 (+1)	200 (0)	7.63	7.61
3	100 (+1)	20 (0)	16 (−1)	200 (0)	5.93	5.92
4	75 (0)	15 (−1)	16 (−1)	200 (0)	6.92	6.88
5	75 (0)	25 (+1)	16 (−1)	200 (0)	7.32	7.24
6	50 (−1)	20 (0)	24 (+1)	200 (0)	6.57	6.62
7	75 (0)	15 (−1)	20 (0)	150 (−1)	6.69	6.70
8	75 (0)	20 (0)	20 (0)	200 (0)	7.64	7.60
9	75 (0)	20 (0)	20 (0)	200 (0)	7.62	7.60
10	100 (+1)	25 (+1)	20 (0)	200 (0)	6.35	6.42
11	75 (0)	25 (+1)	20 (0)	150 (−1)	6.75	6.70
12	75 (0)	20 (0)	20 (0)	200 (0)	7.51	7.60
13	75 (0)	25 (+1)	20 (0)	250 (+1)	7.94	7.97
14	75 (0)	20 (0)	16 (−1)	250 (+1)	7.22	7.17
15	100 (+1)	20 (0)	24 (+1)	200 (0)	6.57	6.50
16	100 (+1)	20 (0)	20 (0)	150 (−1)	5.54	5.53
17	75 (0)	20 (0)	20 (0)	200 (0)	7.69	7.60
18	75 (0)	20 (0)	20 (0)	200 (0)	7.56	7.60
19	50 (−1)	20 (0)	16 (−1)	200 (0)	6.35	6.46
20	100 (+1)	15 (−1)	20 (0)	200 (0)	6.15	6.12
21	75 (0)	15 (−1)	24 (+1)	200 (0)	7.22	7.26
22	50 (−1)	25 (+1)	20 (0)	200 (0)	6.76	6.81
23	75 (0)	15 (−1)	20 (0)	250 (+1)	7.18	7.26
24	50 (−1)	20 (0)	20 (0)	150 (−1)	6.23	6.12
25	75 (0)	20 (0)	16 (−1)	150 (−1)	6.57	6.65
26	50 (−1)	15 (−1)	20 (0)	200 (0)	6.45	6.40
27	75 (0)	20 (0)	24 (+1)	150 (−1)	6.56	6.63
28	75 (0)	20 (0)	24 (+1)	250 (+1)	7.99	7.93
29	50 (−1)	20 (0)	20 (0)	250 (+1)	6.83	6.78

*A*: ethanol concentration, %; *B*: homogenate/ultrasonic time, min; *C*: solvent-to-solid ratio, mL/g; *D*: ultrasonic power, W.

**Table 2:** Analysis of variance (ANOVA) for the fitted quadratic model of *C. camphora* flavonoids extraction determined from Box–Behnken design.

Source	Sum of squares		Degree of freedom		Mean square	*F*-value	*P*-value
Model	10.86		14		0.78	101.79	<0.0001^*∗∗∗*^
*A*	0.33		1		0.33	43.73	<0.0001^*∗∗∗*^
*B*	0.38		1		0.38	49.93	<0.0001^*∗∗∗*^
*C*	0.42		1		0.42	54.81	<0.0001^*∗∗∗*^
*D*	2.49		1		2.49	327.14	<0.0001^*∗∗∗*^
*AB*	0		1		0	0.46	0.5102
*AC*	0.04		1		0.04	5.57	0.0333^*∗*^
*AD*	0.07		1		0.07	8.73	0.0104^*∗*^
*BC*	0		1		0	0	0.9865
*BD*	0.12		1		0.12	16.35	0.0012^*∗∗∗*^
*CD*	0.15		1		0.15	19.75	0.0006^*∗∗∗*^
*A* ^2^	6.74		1		6.74	884.93	<0.0001^*∗∗∗*^
*B* ^2^	0.14		1		0.14	18.29	0.0008^*∗∗∗*^
*C* ^2^	0.29		1		0.29	37.47	<0.0001^*∗∗∗*^
*D* ^*2*^	0.58		1		0.58	76.32	<0.0001^*∗∗∗*^
Residual	0.11		14		0		
Lack of fit	0.09		10		0	1.75	0.3111
Pure error	0.02		4		0		
Cor. total	10.97		28		0.78		
Credibility analysis of the regression equations							
Std. dev.	Mean	CV %	Press	*R* ^2^	Adjusted *R* ^2^	Predicted *R* ^2^	Adequacy precision
0.87	6.91	1.26	0.53	0.9903	0.9805	0.9516	38.782

*A* : ethanol concentration, %; *B* : homogenate/ultrasonic time, min; *C* : solvent-to-solid ratio, mL/g; *D*: ultrasonic power, W; Cor. total: totals of all information corrected for the mean; Std. dev.: standard deviation; CV: coefficient of variation. ^*∗*^ *P* < 0.1, significant; ^*∗∗*^ *P* < 0.01, highly significant; ^*∗∗∗*^ *P* < 0.001, extremely significant.

**Table 3:** Linearization of second-order kinetic model for *C. camphora* flavonoids extraction obtained by HUCSE and HRE.

Method	Equation	Slope	*Y*_*S*_ (mg/g)	Intercept	*h*	*k*	*R* ^2^
HUCSE	*t/Y*_*t*_ = 0.0669 *t* + 1.4724	0.0669	14.95	1.4724	0.68	3.04 × 10^−3^	0.9995
HRE	*t/Y*_*t*_ = 0.0766 *t* + 12.2890	0.0766	13.05	12.2890	0.08	4.70 × 10^−4^	0.9984

## Data Availability

The data used to support the findings of this study are available from the corresponding author upon request.
